# Immunotherapy in head and neck squamous cell carcinoma and rare head and neck malignancies

**DOI:** 10.37349/etat.2021.00062

**Published:** 2021-12-31

**Authors:** Stefano Cavalieri, Daria Maria Filippini, Arianna Ottini, Cristiana Bergamini, Carlo Resteghini, Elena Colombo, Roberta Lombardo, Imperia Nuzzolese, Salvatore Alfieri, Lisa Licitra, Laura D. Locati

**Affiliations:** 1 Head and Neck Cancer Medical Department, Fondazione IRCCS Istituto Nazionale dei Tumori di Milano, via Venezian 1, 20133 Milan, Italy; 2 Department of Oncology and Hemato-Oncology, University of Milan, via Festa del Perdono 7, 20122 Milan, Italy; Università Politecnica Marche, Italy

**Keywords:** Head and neck cancer, immune checkpoint inhibitors, rare cancer

## Abstract

The dismal prognosis of recurrent/metastatic (R/M) head and neck squamous cell carcinoma (HNSCC) prompted recent advances in the field of therapeutic approaches beyond cytotoxic cancer therapy. In recent years, the deeper and increasing knowledge on the genomic landscape and the upcoming new data on immunotherapy enacted by HNSCCs have led to successful therapeutic targeting of the immune system. Immune checkpoint inhibitors (ICIs) have changed state of the art in R/M patients and could have a potential role even in early disease. The purpose of this work is to summarize the role of immunotherapy for R/M HNSCC in clinical practice, with insights about future perspectives. Updated immunotherapy results in other R/M head and neck cancers such as thyroid, salivary glands, nasopharynx, sinonasal cancers, and nuclear protein in testis (NUT) are presented.

## Introduction

Head and neck cancers (HNC) are a heterogeneous group of malignancies, including several histotypes, and head and neck squamous cell carcinoma (HNSCC) is the most frequent. They may arise from the oral cavity, oropharynx, hypopharynx, larynx, and nasopharynx. This latter has a distinct clinical behavior and unique biology related to Epstein-Barr virus (EBV) infection. Another oncogenic virus involved in HNSCC etiopathogenesis is the human papillomavirus (HPV), almost correlated to oropharyngeal cancer (OPC).

HPV-related HNSCCs have a distinct immune landscape compared to HPV-negative cases. The presence of diffuse immune infiltrates including CD8^+^, Tregs, natural killer (NK) cells, B cells, and in HPV-related HNSCC suggest that checkpoint inhibitors may be beneficial for these patients [1, 2]. By contrast, HPV-negative cancers have a lower number of programmed cell death protein 1 (PD-1) expressing CD4^+^ and CD8^+^ T cells than their HPV-positive counterparts [3]. Meanwhile, the existence of a high mutation burden in HPV-negative tumors, characterized by a high degree of smoking-related signatures, is a favorable opportunity for antitumor immunity to tackle the dominant immunosuppressive environment. However, the current results with checkpoint inhibitors in HPV-related cancers are still contradictory.

The landscape of immunotherapy in recurrent/metastatic (R/M) HNSCC has been rapidly evolving in the last years, and many clinical studies have been published so far. In the medical communities, the most famous ones are those that led to the regulatory approval of immune checkpoint inhibitors (ICIs) in clinical practice, but several other studies were negative, failing to provide a survival benefit in HNSCC patients. Moreover, the main achievements have been reached in the R/M setting, but more recently the research has been focusing on loco-regionally advanced potentially curable diseases.

In this review, we summarized the actual ICIs for R/M HNCs in clinical practice, outlining the results of the most recent clinical trials and looking to future perspectives. Even though the vast majority of the immuno-oncological research in HNC has been focused on ICIs, we mentioned also some studies exploring cell-based therapies and new discovery-based approaches. Immunotherapy for thyroid cancer and rare R/M HNCs, such as salivary glands, nasopharynx, paranasal sinus, and nuclear protein in testis (NUT) midline carcinoma is also reviewed.

## Standard of care

The role of ICIs has been mainly investigated in the R/M setting of HNSCC [4, 5], both in platinum-resistant and platinum-sensitive diseases.

### Platinum-sensitive R/M HNSCC

For nearly three decades, the mainstay of first-line palliative systemic chemotherapy (CT) has been cisplatin combined with 5-fluorouracil (5-FU) [6] or, more recently, with taxane [7] due to the increased response rate.

Food and Drug Administration (FDA) approved the anti-epidermal growth factor receptor (EGFR) monoclonal antibody cetuximab in 2006 for patients with HNSCC. When combined with platinum and 5-FU (the so-called “EXTREME” regimen), cetuximab significantly increased both progression-free survival [PFS, hazard ratio (HR) = 0.54, 95% confidence interval (CI): 0.43–0.67] and overall survival (OS, HR = 0.80, 95% CI: 0.64–0.99) in patients with R/M disease representing until 2019 the standard-of-care for R/M HNSCC patients [8]. Alternative cetuximab plus platinum-based CT regimens include cisplatin + cetuximab +/− paclitaxel (the B490 phase II Italian study) [9], and cisplatin + cetuximab + docetaxel (the so-called “TPEx” regimen explored in phase II French study) [10]. Whatever CT is associated with platinum, until 2019, the mainstay treatment for platinum-sensitive R/M HNSCC was a cetuximab + platinum-based CT. With this therapeutic approach, objective response rate (ORR) ranges from 36% (EXTREME) to 59% (TPEx), median PFS from 5.1–5.6 (EXTREME) to 6–7 months (B490), median OS from 10.1–10.7 (EXTREME) to 14.5 months (TPEx). In 2019, based on the results from KEYNOTE-048, both the FDA and the European Medicines Agency (EMA) approved the anti-PD-1 pembrolizumab (pembro) alone or with CT as first-line therapy in R/M HNSCC [11]. FDA-approved pembro with CT regardless of programmed death-ligand 1 (PD-L1) status, and pembro alone for patients with a combined positive score (CPS) PD-L1 ≥ 1. By contrast, EMA approval was reserved for patients with CPS PD-L1 ≥ 1 expressing tumors. So far, PD-L1 was the first predictive biomarker for treatment efficacy with PD-1 inhibitors in HNSCC.

The KEYNOTE-048 trial included 882 platinum-sensitive R/M HNSCC patients randomized 1:1:1 to receive pembro monotherapy, pembro plus cisplatin/carboplatin, and 5-FU *versus* the EXTREME regimen. Pembro significantly prolonged OS in patients with CPS > 1 and ≥ 20; irrespectively of PD-L1, pembro was non-inferior to the EXTREME regimen [12].

ORR and PFS were similar between pembro plus CT and the EXTREME regimen (ORR 36.3% *versus* 35.6%, PFS 5.1 *versus* 4.9 months, respectively). In comparison, a lower ORR was observed in those patients treated with pembro alone compared to patients treated with EXTREME (23.3% *versus* 36.1% for CPS ≥ 20, 19.1% *versus* 34.9% for CPS ≥ 1). In patients treated with pembro monotherapy, prolonged duration of response (DoR) has been observed compared to standard of care (SoC) for patients with CPS ≥ 20 and ≥ 1 (23.4 *versus* 4.2 months, 24.8 *versus* 4.5 months, respectively). The benefit of pembro has been maintained for those patients with CPS 1–19, where the HR for OS demonstrated a slight advantage of pembro *versus* EXTREME and favored pembro + CT over EXTREME. In CPS-negative patients, HR for OS was 1.51 (95% CI: 0.96–2.37) for pembro *versus* EXTREME and 1.21 (95% CI: 0.76–1.94) for pembro + CT *versus* EXTREME, even if the analyses could have been limited by the small sample size of this group of patients [13]. Pembro alone demonstrated a more favorable safety profile with a lower incidence of treatment-related adverse events (TRAEs) equal to or higher than grade 3 TRAEs compared to EXTREME (17% *versus* 69%). No significant differences have been observed between pembro plus CT *versus* EXTREME (71% *versus* 69%). Based on these data, the society for immunotherapy of cancer published a consensus statement on immunotherapy for the treatment of HNSCC, where they suggested the use of a combination of ICI plus CT in patients requiring a quick tumor shrinkage such as those with more significant tumor burden and rapidly growing disease [14].

Despite the outstanding results of KEYNOTE-048, the response rate with ICIs is still modest. To intensify immune response, two studies (CheckMate-651 and Kestrel) are currently testing the efficacy of the combination of anti-cytotoxic T-lymphocyte-associated antigen 4 (CTLA-4) and anti-PD-L1 agents *versus* the EXTREME regimen for first-line R/M HNSCC.

The CheckMate-651 randomized (1:1) 947 patients to combined therapy with the anti-PD-1 nivolumab (nivo) and the anti-CTLA-4 ipilimumab *versus* the EXTREME regimen (cetuximab + 5-FU + cisplatin or carboplatin) [15] independently of PD-L1 status. The primary endpoint of this phase III study is OS in all comers and in PD-L1 positive patients. The final results of this study have been presented at European Society for Medical Oncology (ESMO) 2021 Annual Conference, and have shown that the immunotherapy combination did not provide a statistically significant improvement over EXTREME [15].

The KESTREL study was a randomized trial assessing three interventions: a combination of anti-CTLA-4 tremelimumab + anti-PD-L1 durvalumab *versus* durvalumab alone *versus* standard CT (EXTREME) [16]. In February 2021, a press release announced that the study did not meet its primary endpoint, which was OS improvement.

In conclusion, immunotherapy with the anti-PD-1 pembro (alone or in combination with CT) is the current SoC for PD-L1 + platinum-sensitive R/M HNSCC.

### Platinum-resistant R/M HNSCC

The prognosis is still dismal in loco-regionally advanced HNSCC patients recurring in the first months after platinum-containing curative treatments and in those progressing on platinum-based CT. In this setting, until 2016 the SoC was single agent CT with methotrexate or taxanes (docetaxel or paclitaxel), or with the single agent anti-EGFR cetuximab, especially in the USA. SoC provides a modest ORR (5.8%), and prognosis is poor (median PFS 2.3 months, median OS 5.1%) [5].

In platinum-resistant R/M HNSCC, several studies have explored the efficacy and the safety of ICIs. The two pivotal studies that led to the approval of anti-PD-1 agents pembro and nivo in this setting are the phase III KEYNOTE-040 [4] and CheckMate-141 [5] clinical trials. For nivo, the license from the regulatory agencies was not conditioned by PD-L1 expression. On the contrary, the EMA approved pembro only in those patients with R/M HNSCC expressing PD-L1 with a ≥ 50% tumor proportion score (TPS) and progressing on or after platinum-containing CT.

The main results of CheckMate-141 and KEYNOTE 040 are reported in Table 1.

**Table 1.** Trials of approved checkpoint inhibitors for R/M HNSCC

**Authors**	**Study**	**Setting**	**Study arm**	**Control arm**	**Median OS**		**Approval**
Ferris et al. [5]	CheckMate-141	Platinum-resistant	Nivo	SoC	Nivo: 7.5 months SoC: 5.1 months		FDA EMA
Cohen et al. [4]	KEYNOTE-040	Platinum-resistant	Pembro	SoC	Pembro: 8.4 months SoC: 6.9 months		FDA EMA (TPS ≥ 50%)
Burtness et al. [11]	KEYNOTE-048^*^	First-line	Pembro	EXTREME	*PD-L1* CPS ≥ 20 Pembro: 14.9 months EXTREME: 10.8 months	*PD-L1* CPS ≥ 1 Pembro: 12.3 months EXTREME: 10.4 months	FDA EMA
			Pembro + CT	EXTREME	*PD-L1* CPS ≥ 20 Pembro + CT: 14.7 months EXTREME: 11.1 months	*PD-L1* CPS ≥ 1 Pembro + CT: 13.6 months EXTREME: 10.6 months	

* ESMO 2020 Congress update

The CheckMate-141 design was similar to that of the KEYNOTE-040, except for some main differences such as patient selection, dosages, schedules, and distribution of SoC regimens and treatments beyond progression [17]. The KEYNOTE-040 excluded patients recurring or progressing within three months of previous multimodal therapy containing platinum for locally advanced disease, and only 1.2% (*N* = 6 of 495) of the HNSCC patients were heavily pre-treated (≥ 3 lines of prior systemic therapy) as compared to 19.9% (*N* = 72 of 361) in the CheckMate-141. Among SoC chemotherapies, docetaxel was administered at 75 mg/m^2^ every 3 weeks (Q3W) in the KEYNOTE-040 trial, whereas in CheckMate-141 it was 30 mg/m^2^ QW. Moreover, the fraction of subjects pre-treated with docetaxel and cetuximab differed in the two studies. Those treated with prior docetaxel were 42% patients in the pembro study *versus* 21% in the nivo one, those with cetuximab were 30% *versus* 11%, respectively. By contrast, in the CheckMate-141, there was a higher number of patients who received methotrexate as SoC (38% *versus* 27% in KEYNOTE-040). Moreover, the percentage of patients in the SoC arm treated by ICIs beyond progression was different. Indeed, in the second-line pembro trial, an ICI was administered at disease progression in 12.5% of patients included in the SoC arm, potentially impacting on OS of these patients.

Other clinical trials studying different ICIs failed to demonstrate a significant improvement in the management of R/M HNSCC. The most relevant ones are the HAWK, CONDOR, EAGLE, and CheckMate-714.

The HAWK was a phase II single-arm trial assessing the activity of the anti-PD-L1 durvalumab in R/M HNSCC platinum-resistant patients with PD-L1 high expression (defined as patients with ≥ 25% of tumor cells expressing PD-L1). It included 112 patients, and the ORR of durvalumab was 16.2% (95% CI: 9.9–24.4), with higher responses in HPV-positive patients (29.4% *versus* 10.9% HPV-negative). Minor safety issues were observed (8% grade 3 TRAEs). Survival was in line with that already described in other single-agent immunotherapy (median PFS and OS 2.1 and 7.1 months, respectively) [18].

The CONDOR trial included platinum-resistant PD-L1 negative R/M HNC patients. This was a phase II open-label study enrolling 267 patients that were randomized in 3 arms: 2 were similar to the KESTREL ones (durvalumab + tremelimumab; durvalumab), the third one explored anti-CTLA-4 alone (tremelimumab). The primary endpoint was ORR. The addition of tremelimumab failed to result in an increased ORR: 9.2% with durvalumab, 1.6% with tremelimumab, and 7.8% with the combination. This modest response rate may be explained by the low expression of PD-L1, but the survival benefit was similar to what is usually reported in second-line treatments with anti-PD-1 [19]. The rate of grade 3–4 TRAEs was similar in three arms with 15.8%, 12.3%, and 16.9%, respectively.

The EAGLE was a randomized, open-label, phase III trial that included 736 platinum-resistant patients to receive second-line durvalumab alone, durvalumab + tremelimumab, or SoC (primary endpoint OS). Unfortunately, no increase in survival was observed. However, outcomes were similar to the ones found with anti-PD-1 agents nivo and pembro. Several reasons have been claimed to interpret the negative results. Patients with PD-L1 expressing tumor could be potentially under-represented in the durvalumab arm; tremelimumab is an IgG2 monoclonal antibody anti-CTLA-4 and, as such, unable to induce the antibody-dependent cell cytotoxicity; moreover, we could not exclude a different activity of PD-1 and PD-L1 agents. Furthermore, it seems that the SoC arm has been somehow favored by the enrolment of more patients with Eastern Cooperative Oncology Group (ECOG) performance status (PS) 0 and with the checkpoint therapy administered in 36% of patients beyond progression [20]. Interestingly, peripheral blood tumor cell mutation burden (bTMB) proved to be a prognostic factor [21]. Based on that, bTMB has become the second predictive marker along with PD-L1 expression for the activity of checkpoint inhibitors.

The phase II randomized trial CheckMate-714 explored the anti-PD-1 nivo +/− the anti-CTLA-4 ipilimumab, with ORR as the primary endpoint [22]. Although final results are still pending, a press release from Bristol-Myers Squibb anticipated that this trial did not meet its primary endpoint.

The negative results of CONDOR, EAGLE, and CheckMate-714 trials hinted at the possible limitation of the activity of anti-CTLA-4 for platinum-resistant R/M HNSCC patients. At this stage, anti-PD-1 monotherapy with nivo or, where approved, pembro is indicated in this patient population as a single agent.

In conclusion, immunotherapy with the anti-PD-1 nivo or pembro is the current SoC for platinum-resistant R/M HNSCC, independently of PD-L1 expression.

## Discovery-based and a new generation of immune-oncology agents

Different strategies have been hypothesized to improve the activity of ICI in HNCs, including approaches tackling tumor microenvironment (TME) and host factors. Indeed, despite a large number of trials, the global activity of immunotherapy is still weak and limited to a very selected patient population (PD-L1 positive and bTMB > 16 mutations/megabase) because of primary and secondary resistance. HNCs microenvironment is characterized by an immunosuppressive feature, and the early angiogenic switch is one of the significant contributors to the accumulation of immune suppressive cells in the TME [2]. Cancer growth is associated with hypoxia that, in turn, induces the production of vascular endothelial growth factor (VEGF) and cytokines [e.g., interleukin 1 (IL-1), IL-6, and IL-17]. VEGF binding to VEGF receptor 2 (VEGFR2) on Treg cells and myeloid-derived suppressor cells (MDSCs) participates in the maintenance of the immunosuppressive TME. Moreover, the hypoxic status contributes to the impairment of T-cell effectors and PD-L1 upregulation, providing a solid rationale in combining antiangiogenic agents with ICI. In this context, the association of lenvatinib (a multi-kinase inhibitor with a significant antiangiogenic activity) plus pembro is one of the most investigated in several malignant tumors. In a phase Ib/II trial, this association in unselected, heavily pretreated patients with multiple solid tumors resorted to an ORR of 36.4% in R/M HNSCC according to immune-related response evaluation criteria in solid tumors (RECIST) [23] with 91% of patients experienced a side effect higher than grade 3 [24]. These toxicities included adverse events (AEs) more specifically associated with antiangiogenic agents (e.g., arterial hypertension). However, some of the observed toxicities were known to be both immune-related and antiangiogenic-related (e.g., fatigue and diarrhea).

Based on this promising activity, a prospective phase III study is currently recruiting (LEAP-010) patients with R/M HNSCC, PD-L1 CPS ≥ 1, to receive pembro plus lenvatinib 20 mg or placebo first-line [25]. Several trials using the same approach are ongoing in R/M HNSCC (e.g., phase II trials with the anti-PD-1 pembro and ramucirumab; anti-PD-L1 atezolizumab plus bevacizumab) [26, 27].

The former study is made of two parts: the first is a de-escalation phase I trial of the combination of the anti-VEGFR2 monoclonal antibody ramucirumab + the anti-PD-1 pembro; in case of absence of safety concerns, and after defining the optimal drug dose, a second phase will be a single-arm phase II trial assessing the aforementioned combination [24]. The ATHENA study is a phase II trial exploring the activity and safety of the association of the anti-PD-L1 atezolizumab and the anti-VEGF-A/B monoclonal antibody bevacizumab in platinum-resistant R/M HNSCC [25]. In both studies, the primary endpoint is the ORR.

Other molecular actors of the TME are the focus of the current clinical research. One of the most relevant includes indoleamine deoxygenase (IDO), involved in tryptophan metabolism [28], contributing to the immunosuppressive features of the TME. IDO1 is targeted by epacadostat. Within the KEYNOTE-037/Echo-202 phase I/II trial, epacadostat plus pembro determined a stable disease (SD) as the best response in one HNSCC patient [29]. In this context, the association of epacadostat + pembro is under investigation in the KEYNOTE-669/Echo-304 study [30]. This is a phase III trial comparing the combination of epacadostat + pembro *versus* pembro alone *versus* the EXTREME regimen in the first-line setting. The estimated study completion date is July 2021. Although several studies have been put on hold due to the negative results from the phase III trial in melanoma, in principle, IDO1 remains a very promising immunotherapeutic target. Tumor-associated macrophages (TAMs) [31] may be a crucial target for immunotherapy. They are in the hypoxic area of the tumor where they release VEGF and transforming growth factor-β (TGF-β) that, in turn, attract more TAMs, amplifying this immunosuppressive loop. TAMs phenotype shapes their role within TME, with “M1-like macrophages” acting with pro-inflammatory activities and antitumor effect through IL-12, IL-23, and interferon (IFN) gamma. On the opposite, “M2-like macrophages” provide an immunosuppressive TME with inhibition of “M1-like macrophages”, secretion of IL-10, TGF-β, VEGF, tumor necrosis factor alpha (TNF-α), and induction of angiogenesis.

Inhibiting the colony-stimulating factor (CSF) 1 receptor (CSF1R) can inactivate TAMs, so leading to a more favorable anti-cancer TME. Indeed, CSF1R, the receptor of the soluble mediator CSF, is one of the main actors in determining M2 macrophage polarization [32]. However, the study exploring the CSF1R inhibitor PLX3397 was terminated early owed to the lack of evidence of clinical efficacy [33]. The immunosuppressive feature of TME is shared with other non-HNSCC solid tumors. Besides, the enrichment of specific cells population such as infiltrating NK, CD8^+^ T cells, and Tregs is characteristic of HNSCC TME. In fact, human leucocyte antigen (HLA)-E is overexpressed in HNSCCs, and it acts inhibiting NK and CD8^+^ T cells via NK group 2 member A (NKG2A), paving the way to tailored immunotherapeutic approaches. Monalizumab is an ICI targeting NKG2A, which is expressed on subsets of NK. NKG2A blockade promotes immune response mediated by NK and CD8^+^ T cells, and when combined with cetuximab, it enhances NK cell antibody-dependent cell-mediated cetuximab-induced cytotoxicity by itself. The effective combination of monalizumab and cetuximab has been investigated in a multicentric phase Ib-II trial [34]. The results of the phase II cohort on 40 platinum-resistant patients showed an ORR of 27.5%, with median PFS and OS of 4.5 and 8.5 months, respectively. A very promising ORR of 20% (95% CI: 11–35) was reported in cohort II, including 40 platinum-resistant patients previously treated with PD-L1 inhibitors. PFS and OS were still immature at the time of data presentation [35]. Based on these results, the INTERLINK-1 phase III study [36], has recently started, comparing monalizumab plus cetuximab *versus* placebo plus cetuximab in patients with R/M HNSCCs who had received a prior ICI and platinum-based CT but not cetuximab. Survival is the primary aim.

In addition to the block of negative regulatory receptors, the enhancement of co-stimulatory signals using agonistic monoclonal antibodies and small molecules is under evaluation. For example, the benefit of feladilimab [GlaxoSmithKline 3359609 (GSK3359609)], an inducible T-cell co-stimulator (ICOS) agonist added to pembro was demonstrated in INDUCE-1 [37] with an ORR of 24%, a disease control rate (DCR) of 68%: median PFS was 4.2 months, median OS 13.1 months [38].

The INDUCE-3 was a randomized, double-blind, adaptive, phase II/III study of feladilimab or placebo in combination with pembro for first-line treatment of CPS ≥ 1 R/M HNSCC [39]. The primary study endpoints were OS and PFS. In April 2021, the trial sponsor communicated that a lack of benefit in terms of efficacy was observed in the first interim analysis. For this reason, GSK decided to stop the enrolment in the INDUCE-4 trial as well. This was a randomized, double-blind, adaptive, phase II/III study of feladilimab in combination with pembro and 5-FU-platinum CT *versus* placebo in combination with pembro plus 5-FU-platinum CT for first-line treatment of R/M HNSCC [40].

Further immune checkpoints have been recently selected as potential targets for therapeutic agents, mostly in combination with other already available drugs. Most of them are co-stimulatory molecules involved in immune response, so there is a solid rationale to explore the clinical activity of their inhibition. Among them there are lymphocyte-activation gene 3 (LAG3) [41], which is targeted by relatlimab [42, 43] and eftilagimod [44]; ITIM domain (TIGIT) [45], that is inhibited by tiragolumab, a drug under investigation in combination with anti-PD-L1 atezolizumab as first-line treatment for PD-L1 positive R/M HNSCC [46]; TNFR-related gene (GITR) [47], whose agonist is under investigation in a phase Ib trial of multivalent autophagosome vaccine with anti-PD-1 immunotherapy [48].

In the last years, the employ of bi-specific antibodies has been considered as well. They are usually made of one subunit that is active against two different molecular targets. The most relevant are mucin domain 3 (TIM3) [49], tackled by the PD-1/TIM3 bispecific antibody RO7121661 [50] and TGF-β, a well known immunosuppressive mediator [51], that is co-inhibited by bintrafusp alfa, an anti-TGF-β/anti-PD-1 agent that showed promising activity in HNSCC [52]; IL-2 [53] and fibroblast-activating protein (FAP) [54] are the targets of RO6874281, a bispecific cytokine acting on tumor-associated fibroblasts, that induced one durable response in one HNSCC patient included in a phase I study [55].

Therapeutic vaccination is another strategy currently in use to enhance immune response. Whether HPV-related HNSCC viral antigens (e.g., E6/E7) represent a potential target for vaccination, for HPV-negative HNSCCs tumor associated-antigens (TAA) such as for example mucin 1 (MUC1), carcinoembryonic antigen (CEA), and melanoma-associated antigen-A3 (MAGEA3, unmutated antigens) should be considered [56–59]. Tumor-specific antigens (TSA), deriving from cancer driver mutations [e.g., tumor protein p53 (TP53), rat sarcoma (RAS)] or passenger mutations, are under exploitation for clinical benefit [60]. ISA101 is the most advance in clinical development in HPV-related HNSCCs. This is a synthetic long-peptide HPV-16 vaccine inducing HPV-specific T cells in patients with incurable HPV-16-related cancers [61]. In the ISA101-MDACC study combining ISA101 and the PD-1 inhibitor nivo, the ORR was 30% in subjects with HPV-16 positive OPC [62]. A promising association of anti-PD-1 and ISA101 is under current evaluation in a randomized, double-blind, placebo-controlled, phase II trial that is testing the combination of the ISA101b vaccine and cemiplimab in HPV-16-positive R/M OPC patients [63]. The same vaccine is under evaluation in combination with utomilumab (anti-CD137 immunostimulatory antibody) [64]. Several trials testing therapeutic vaccination in a different phase of development both in HPV-related and HPV-unrelated HNSCCs are recruiting [65].

**Figure 1.**
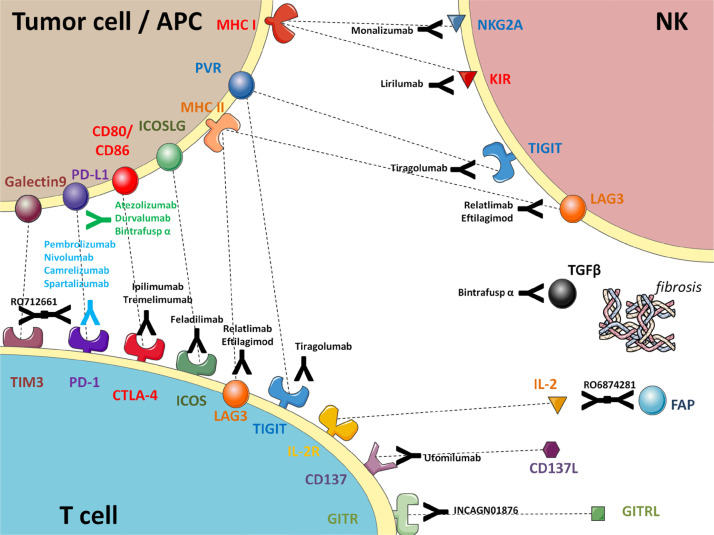
Schematic summary of approved and experimental immunotherapy agents in HNSCC (prepared using the open-access graphical repository of SMART-servier medical art). APC: antigen-presenting cell; MHC: major histocompatibility complex; PVR: poliovirus receptor; GITRL: the ligand for glucocorticoid-induced TNFR family-related protein; KIR: killer cell immunoglobulin-like receptors; IL2R: IL-2 receptors; ICOSLG: inducible T-cell costimulator ligand

To provide a general overview of the several ICIs cited in this article, Figure 1 summarizes the mechanisms of action of some of them. In conclusion, some of the most recent ICIs (e.g., anti-ICOS) failed to improve the outcomes of R/M HNSCC over SoC immunotherapy. However, there are many innovative approaches targeting the tumor immune microenvironment that are still under investigation, and some of them have shown promising activity.

### Cell-based therapy: chimeric antigen receptor T cell and adoptive T-cell therapies

Chimeric antigen receptor (CAR) T cell (CAR-T) therapy is based on T cells expressing an engineered receptor for a specific cancer antigen. CAR-T has been approved for B-cells malignancies such as mantle cells lymphoma, acute lymphoblastic leukemia, and large B cell lymphoma. This therapy is based on genetically modified autologous T cells expressing a CD19-specific CAR, lysing CD19-positive targets (normal and malignant B lineage cells).

The efficacy of CAR-T in solid tumors, particularly in HNCs, has to be still proved. Indeed, the main issues in this context are to find the suitable tumor antigen to tackle as CD19 for the hematological malignancies. For example, dysregulation of ErbB signaling has been involved in the pathogenesis of HNCs, potentially providing a candidate target for CAR-T therapy. A phase I/II trial with a gene-modified cellular therapy, called T4 immunotherapy, is currently ongoing in HNCs. These cells are engineered to co-express two transgenes: a CAR, T1E28z, targeted against a range of ErbB homodimers and heterodimers and a chimeric cytokine receptor, 4αβ, that allows the selective *ex vivo* expansion of engineered cells using IL-4. To limit the risk of side effects and in contrast to standard CAR-T therapy, engineered T-cells were delivered intratumor without prior lymphodepletion [66]. Preliminary data in 13 patients seem to confirm that intratumor administration of T4 immunotherapy is safe, being all TRAEs observed below or equal to grade 2, with no dose-limiting toxicities [common terminology criteria for adverse events (CTCAE) v4.0]. Despite the disease progression at study entry, an SD was observed in all 13 enrolled patients with the dosage ≥ 10 × 10^7^ T4^+^ T-cells [67]. A clinical trial involving patients with HPV-related head and neck and cervical cancer is currently recruiting [68]. The study tests engineered CAR-T that recognize the E6 HPV antigen. Twenty patients will be randomized to receive HPV-E6-specific CAR-T cells with or without anti-PD-1. Cross talk between PD-L1/PD-1 induces T cell exhaustion, and CAR-T cells armed with a PD-1 antagonist could potentially enhance the efficacy of CAR-T.

Different approaches with adoptive T-cell therapy have been investigated in EBV-related nasopharyngeal cancer (NPC). EBV-specific autologous polyclonal cytotoxic T lymphocytes (CTL) were administered with significant clinical and immunological responses in patients with relapsed/metastatic EBV-related NPC [69–72]. More recently, a clinical trial with CAR-T cells involving both NPC and breast cancer patients with epithelial cell adhesion molecule (EpCAM) CAR-T has been initiated. The study is currently still recruiting [73].

Only preclinical data on CAR-T have been published in salivary gland [74] and thyroid carcinomas [75].

As a general statement, the situation of CAR-T cells is much less advanced in HNSCC than in other malignancies (e.g., onco-hematologic ones). The recruitment of the ongoing studies still needs to be completed, and we anticipate that for at least some years HNC patients will not benefit from these innovative approaches, at least in clinical practice.

## Advanced thyroid and rare head and neck cancers

The studies cited in previous paragraphs were conducted in R/M HNSCCs. In this setting, first-line pembro has become the first option for PD-L1 positive (CPS ≥ 1) disease, and second-line nivo is the best option for platinum-resistant R/M HNSCC. However, the pivotal trials that led to the regulatory approval of these ICIs did not include thyroid cancers and rare HNC. Since healthcare professionals devoted to HNC can face not only HNSCC, but also these tumor types, the following sections will concentrate on the role of immunotherapy in thyroid cancer and in salivary gland, nasopharyngeal, and paranasal sinuses carcinomas.

### Thyroid cancer

Thyroid cancer (TC) can be classified in differentiated TC (DTC, including papillary TCs and follicular TCs: PTCs and FTCs, respectively), medullary TC (MTC), and anaplastic TC (ATC). DTC is usually treated with surgery and radiometabolic treatment. In radioactive-iodine (RAI) refractory DTC, the current SoC is the antiangiogenic multi-kinase inhibitor lenvatinib. With this drug, the ORR, median PFS, and median OS are 64.8%, 18.3 months, and not reached respectively [76].

Recently, at American Society of Clinical Oncology (ASCO) 2021, the results of the COSMIC-311 phase III randomized trial showed the superiority of cabozantinib over placebo after lenvatinib failure in RAI-refractory DTC [77]: ORR was 15% (*versus* 0% with placebo), and median PFS and OS were not reached.

MTC is a neuroendocrine malignancy arising from the parafollicular C cells of the thyroid. In the non-operable or metastatic setting, standard treatments include the multi-kinase antiangiogenic inhibitors vandetanib and cabozantinib [78]. The latter provides a significant benefit, especially in MTCs harboring rearranged in transfection (RET) M918T mutation (ORR 34%, median PFS 13.9 months, median OS 44.3 months) [79].

ATC is an aggressive disease, often not operable, with a dismal prognosis. Recently, the combination of B type rapidly accelerated fibrosarcoma oncogene (BRAF) and mitogen-activated protein kinase (MEK) inhibitors showed to be active and safe in BRAF V600E advanced ATC (ORR 69%, 12-month PFS 79%, 12-month OS 80%) [80].

Unselected TC patient population seems to have a weak benefit from immunotherapy, except for more aggressive DTC histotypes and ATC. Indeed, the safety and the antitumor activity of pembro 10 mg/kg Q2W were demonstrated in patients with PD-L1-positive, advanced PTC, and FTC who were enrolled in the multi-cohort phase Ib KEYNOTE-028 trial. Confirmed partial response (PR) was observed in 2 patients with PTC out of 22 enrolled, for an ORR of 9% (95% CI: 1–29%) with a median response duration of 20 and 8 months, respectively. Global clinical benefit rate (i.e., confirmed PR + SD ≥ 6 months) was 50% (95% CI: 28–72%) [81].

Among the different strategies enacted to improve the activity of PD-1/PD-L1 axis inhibitors, combination with other classes of agents (e.g., antiangiogenic compounds) or with anti-CTLA-4 is under evaluation. Lenvatinib 20 mg plus pembro 200 mg i.v. Q3W upfront in patients with radioiodine-refractory (RAIR) progressive DTCs has been recently reported at ASCO 2020. Eighteen (64%) out of 28 evaluable patients had a PR, 32% had SD. An overlapping PR rate (63%) was reported with single-agent lenvatinib in the randomized trial [82], suggesting the lack of an addictive effect as upfront treatment, although patients were enrolled regardless of PD-L1 expression. The clinical benefit rate [complete response (CR) + PR + SD] was 96%. The primary aim was not met (CRs rate), and 12-month PFS was 75%, appearing favorable compared to single-agent lenvatinib (63%) and pembro (36%). The side effect profile was not negligible, with 83% of patients experiencing grade 3 and 4 toxicities [83]. This study intended to investigate salvage pembro in patients undergoing disease progression (PD) during lenvatinib. PR was 17% with 11 months of PFS [7.1-not estimable (NE)], suggesting a role of pembro in overcoming lenvatinib resistance [84].

The combination of nivo 3 mg/kg Q2W plus ipilimumab 1 mg/kg Q6W was investigated, but it failed to meet the expectations in a series of unselected patients with RAIR-DTC (*N* = 37), MTC (*N* = 10), and ATC (*N* = 7) [85]. The ORR was 9.4% (95% CI: 2–25%). A higher activity has been observed in Hurtle cell carcinoma and ATC patients. Indeed, anti-PD-1 agent spartalizumab alone demonstrated a RECIST ORR of 19% (95% CI: 8.6–34.1%) in a series of unselected, heavily pretreated ATC patients. According to immune-related RECIST (irRECIST), the ORR was 24% (95% CI: 12.1–39.5%), including three patients with a CR and seven with a PR. As expected, considering the poor prognosis of this tumor, the median PFS was 1.7 months. Median OS was 5.9 months (95% CI, 2.4 months-not reached), with 40% of patients alive at one year. Interestingly, a correlation with a better response and longer PFS and OS was reported in patients with a PD-L1 expression with median OS not yet reached in the subset of patients with PD-L1 > 1% [86]. While the activity of spartalizumab alone in BRAF-mutated ATC patients was weak with an ORR of 8% [87], the association of vemurafenib and cobimetinib with the anti-PD-L1 atezolizumab led to a significant OS increase at 12-months (median OS not yet reached, 95% CI: 15.96-NE) with an ORR of 53% including one patient with CR (6%), supporting the employ of the triplets in BRAF mutated patients population [88]. Trials are currently ongoing to test the role of immunotherapy in advanced thyroid cancers [89]. Recently, at ASCO 2021 annual meeting Leboulleux and colleagues reported the results of the AcSé phase II study [90], a French multicenter clinical trial exploring the activity and safety of pembro in rare malignancies. In the RAI-refractory TC cohort, the ORR was 11.1% in DTC and 18.8% in ATC [90]. The authors concluded that such an ORR was “low in DTC and not negligible in ATC, but with a short DoR”. Further studies are needed to assess the potential added value of immunotherapy to targeted therapy in advanced TC.

In conclusion, for DTC and MTC targeted therapies are the current SoC, and it is unlikely that immunotherapy alone will replace them. On the contrary, for ATC the introduction of immunotherapy has led to a relevant benefit, complementing the one provided by BRAF and MEK inhibition.

### Salivary gland carcinomas

Salivary gland carcinomas (SGCs) have a complex classification, including more than 20 distinct tumor types [91]. The treatment of R/M SGC mainly depends on the histology. As a general approach, it might be helpful to distinguish between adenoid cystic carcinoma (ACC) and non-ACC. The former usually undergo a watchful-waiting strategy or from loco-regional procedures when oligometastatic or paucisymptomatic (e.g., lung metastasectomies [92] or liver local therapies [93]). Symptomatic R/M ACC or cases with a high disease burden can be treated with the multi-kinase inhibitor lenvatinib (ORR 11.5–15.6%, median PFS 9.1–17.5 months, median OS 27.5 months) [94, 95]. Non-ACCs include a very heterogeneous group of malignancies, and the description of the treatment of each of them is outside the topic of the current review. As a general concept, non-ACC are treated with cytotoxic CT (e.g., cisplatin and doxorubicin or carboplatin and taxanes), but chemo-free approaches can be envisaged when specifically druggable molecular alterations are found [96]. Some examples include combined androgen blockade for androgen-receptor overexpression (ORR 64.7%, 3-year PFS 11.8%, 5-year OS 19.3%) [97], trastuzumab (ORR 70.2%, median PFS 8.9 months, median OS 39.7 months) [98] or trastuzumab-emtansine (ORR 90%), or trastuzumab-deruxtecan (ORR 47%, median PFS 14.1 months) [99] with human epidermal growth factor receptor 2 (HER2) amplification (both can be found in salivary duct carcinoma (SDC), and in adenocarcinoma not otherwise specified), entrectinib or larotrectinib for tropomyosin receptor kinase (TRK) translocations (mammary-analog secretory carcinoma), etc.

In SGCs, a close correlation between histotype and immunologic microenvironment features has been recently demonstrated [100]. SDC has the most favorable immunogenomic profile, with a moderate tumor mutational burden (TMB) and moderate-to-high levels of microenvironmental inflammation. In contrast, ACCs have immunogenomic profiles that are less favorable for immunotherapy, with very low TMB and an immune-depleted microenvironment. Myoepithelial carcinomas had an intermediate immune profile. These differences might be exploited for a more personalized approach. ICIs are deemed to have limited activity in ACC, where PD-L1 is not expressed in contrast with the overexpression of PD-L2. R/M ACCs have a marked enrichment of several gene alterations, and distinct patterns of intratumoral genetic heterogeneity were observed [101], and few data about the activity of immune checkpoint blockade in SGCs are available. In this setting, the KEYNOTE-028 phase Ib trial included a small SGC cohort of 28 PD-L1 positive patients treated with P. The response was primarily observed in histotypes with a high PD-L1 expression, and higher mutational load, 3 PRs (11.5%) have been reported in two adenocarcinomas nitric oxide synthase (NOS) and one case of high-grade serous carcinoma [102].

More recently, results from a multicentric retrospective study on 24 R/M SGCs treated with nivo showed ORR 4.2%, but some patients achieved long-term disease control and a median PFS of 1.6 and median OS of 10.7 months. A biomarker analysis revealed significantly increased OS in patients with ECOG PS of 0, low neutrophil-to-lymphocyte ratio, lactate dehydrogenase, and C-reactive protein [103].

At ASCO 2021 annual meeting, Burman et al. [104] presented the final results of a phase II trial assessing the activity of nivo + ipilimumab in R/M SGCs. This study included non-ACC patients only and met its primary endpoint of improving the ORR. With combo-immunotherapy, ORR was 16%, and the authors stated that the responses were durable. However, this benefit was observed only in high-grade non-ACC cases.

As in TC, also in SGC targeted therapies have been playing an increasing role. Further research needs to be conducted to confirm the role of immunotherapy in R/M SGCs.

### Nasopharyngeal carcinoma, paranasal cancers, NUT midline carcinoma

NPC is distinct from other HNSCC due to the etiological differences (Epstein Barr virus-related in endemic areas) and its unique biological characteristics [105]. NPC is often diagnosed as a loco-regionally advanced disease, and in this setting, concomitant chemo-radiation is the mainstay treatment. Loco-regional treatments might be preceded or followed by induction or adjuvant CT, respectively [106]. In the R/M setting, the first-line therapy in the last five years has been cisplatin + gemcitabine. This combination CT provides an ORR of 64.1%, and median PFS and OS are 7 and 29.1 months [107].

Preliminary results of a multi-institutional real-world experience of R/M NPC patients showed that ICIs have higher activity in EBV-related cancers [108]. EBV-related NPC cells expressed several viral proteins such as the EBV nuclear antigen 1 (EBNA1), latent membrane protein 1 (LMP1), LMP2A, and LMP2B [109]. These proteins induce a balanced viral replication to maintain the latency of viral infection. At the same time, this phenomenon reduces the possibility of the presentation of viral antigens to the immune system. Moreover, increased PD-L1 expression was associated with LMP1 [110].

Multiple clinical trials have demonstrated an ORR of about 20–25% in patients with platinum-refractory NPC with the single-agent activity of PD-1 inhibitors [111–113].

In a phase II study [114], nine out of 45 enrolled patients with R/M NPC received nivo with an ORR of 20.5%, 1-year OS and PFS were 59%, and 19.3%, respectively. No relevant associations were found with PD-L1 expression, plasma EBV-DNA clearance, and survival. Patients with loss of either or both HLA-A or HLA-B expression had improved PFS [111].

Additionally, results from two single-arm, phase I trials examined camrelizumab, a PD-1 inhibitor, in the treatment of advanced solid tumors and R/M NPC alone and associated with CT. Safety and preliminary antitumor efficacy were reported for camrelizumab monotherapy in a dose-escalation phase I study, which enrolled subjects with advanced solid tumors who had failed current standard antitumor therapies [112]. This open-label, non-randomized, phase I study, evaluated the safety and efficacy of using the combination treatment of camrelizumab, gemcitabine, and cisplatin in R/M NPC [112]. In the camrelizumab monotherapy arm, with a median follow-up of 9.9 months, 34% (95% CI, 24–44%) of evaluable patients experienced a confirmed objective response. Toxicities profile was acceptable, with 16% of patients experiencing TRAEs of grade 3 or 4. For the combination arm, 91% (95% CI, 72–97%) of evaluable patients achieved an objective response, with a severe concern about the safety profile with 87% of patients developing grade 3 TRAEs and 22% grade 4 TRAEs. This toxicity was mostly chemo-related since it was hematological. However, some immune-related AEs (e.g., skin rash) were observed as well, even though less frequently.

The CAPTAIN study, a phase II trial presented at ESMO Virtual Congress 2020, assessed the safety and activity of camrelizumab in R/M NPC who had progressed after more than two lines of CT, with an ORR of 28.2% (95% CI: 21.3–36.0), median PFS and OS of 3.7 and 17.1 months, respectively. The safety profile was manageable, with toxicities consistent with previous reports with camrelizumab [113].

Recently, Yang et al. [115] reported the results of the CAPTAIN-1st study, a multicenter, randomized, double-blind, phase III trial assessing the combination of cisplatin + gemcitabine + camrelizumab *versus* cisplatin + gemcitabine + placebo in R/M NPC. This study showed a relevant improvement of PFS with camrelizumab (median PFS 10.8 months *versus* 6.9 months with placebo, *P* < 0.0001), with a significant ORR (88.1%). The impressive results of the CAPTAIN-1st trial could pave the way for a new first-line treatment for R/M NPC, which shall be chemo-immunotherapy.

Sinonasal epithelial cancers (SNCs) are treated with multimodal treatments, including induction CT and loco-regional approaches [116]. R/M SNC has a poor prognosis (median OS 13 months) [117], and it is usually treated with cytotoxic CT.

Immunotherapy could have a potential role in SNCs, due to its strong association, specifically in undifferentiated sinonasal cancers (SNUC), with immune system-related functional pathways which have been found to contain a high proportion of CD8^+^ effector memory cells in their microenvironment. The identification of immune pathways should be further investigated, for possible integration of immunotherapy into the multidisciplinary approach to these cancers [118], because no data are available at the moment on the activity of checkpoint inhibitors in clinical practice.

NUT carcinoma (or NUT midline carcinoma) is a rare and aggressive subtype of epithelial carcinoma, defined by the evidence of NUT gene (NUTM1) rearrangement. Common sites include the head and neck region (in particular the nasal cavity and paranasal sinuses) and mediastinum [119, 120].

No clinical evidence in the literature has been reported yet about a possible role of ICIs in this setting; however, the efficacy is under evaluation within clinical trials in other sites of the same histology. For instance, the CHANCE trial is aiming to explore the antitumor activity and the safety profile of atezolizumab in pretreated advanced non-small cell lung cancer (NSCLC) patients with rare histological subtypes, including lung NUT-carcinomas [121].

## Conclusion

The landscape of immunotherapy in HNSCC, NPC, SGC, and TC is constantly evolving. This review summarizes the main trial results in these malignant tumors.

In the context of R/M HNSCC treatment, checkpoint inhibitors have changed the state of the art, finally improving patients outcome, even if only a minority of HNSCC patients may benefit from immunotherapy. In the loco-regionally advanced setting, the role of ICIs, especially in combination with radiotherapy or as neoadjuvant/adjuvant agents for surgery is still under study.

To date, the only predictive factors are PD-L1 expression and TMB, and other biological markers are under investigation. The field of research is currently focused on, bringing the benefits of ICI to a more significant portion of patients including rare HNC histotypes. Indeed, promising results have already been reported in SGC, NPC, and TC.

## Abbreviations

5-FU: 5-fluorouracil
ACC: adenoid cystic carcinoma
ASCO: American Society of Clinical Oncology
ATC: anaplastic thyroid cancer
BRAF: B type rapidly accelerated fibrosarcoma oncogene
bTMB: blood tumor cell mutation burden
CAR: chimeric antigen receptor
CAR-T: chimeric antigen receptor T cell
CI: confidence interval
CPS: combined positive score
CR: complete response
CSF: colony-stimulating factor
CSF1R: colony-stimulating factor 1 receptor
CT: chemotherapy
CTLA-4: cytotoxic T-lymphocyte-associated antigen 4
DTC: differentiated thyroid cancer
EBV: Epstein-Barr virus
EMA: European Medicines Agency
ESMO: European Society for Medical Oncology
FDA: Food and Drug Administration
HLA: human leucocyte antigen
HNC: head and neck cancers
HNSCC: head and neck squamous cell carcinoma
HPV: human papillomavirus
HR: hazard ratio
ICIs: immune checkpoint inhibitors
ICOS: inducible T-cell co-stimulator
IDO: indoleamine deoxygenase
IL-1: interleukin 1
LMP1: latent membrane protein 1
MHC: major histocompatibility complex
MTC: medullary thyroid cancer
nivo: nivolumab
NK: natural killer
NKG2A: natural killer group 2 member A
NPC: nasopharyngeal cancer
NUT: nuclear protein in testis
OPC: oropharyngeal cancer
ORR: objective response rate
OS: overall survival
PD-1: mprogrammed cell death protein 1
PD-L1: programmed death-ligand 1
pembro: pembrolizumab
PR: partial response
PS: performance status
PTCs: papillary thyroid cancers
Q3W: every 3 weeks
R/M: recurrent/metastatic
RAI: radioactive-iodine
RECIST: related response evaluation criteria in solid tumors
SD: stable disease
SDC: salivary duct carcinoma
SGCs: salivary gland carcinomas
SoC: standard of care
TAMs: tumor-associated macrophages
TC: thyroid cancer
TGF-β: transforming growth factor-β
TMB: tumor mutational burden
TME: tumor microenvironment
TPEx: cisplatin + cetuximab + docetaxel
TRAEs: treatment-related adverse events
VEGF: vascular endothelial growth factor

## Declarations

### Copyright

© The Author(s) 2021.
